# Transcriptomic analysis offers deep insights into the *Increased Grain Length 1* (*IGL1*) regulation of grain length

**DOI:** 10.1186/s12870-025-06279-2

**Published:** 2025-02-27

**Authors:** Liran Sang, Ending Xu, Yan Liu, Tiange Hu, Mengqi Yang, Jiayu Niu, Chong Lu, Yi Zhou, Yifei Sun, Zhaoyu Zhai, Dina Abdulmajid, Peijiang Zhang, Qianqian Wang, Honggui La, Yu Zou

**Affiliations:** 1 College of Life Sciences, Nanjing Agricultural University, Nanjing , Jiangsu, 210095 China; 2 Anhui Province Key Laboratory of Rice Genetics and Breeding, Rice Research Institute, Anhui Academy of Agricultural Sciences, Hefei, Anhui 230041 China; 3 College of Artificial Intelligence, Nanjing Agricultural University, Nanjing, 210095 China; 4 Rice Research and Training Centre, Field Crops Research Institute, A.R.C, Sakha, Kafrelsheikh 33717 Egypt; 5 Department of Breeding, Shandong Peanut Research Institute, Qingdao, 266000 China

**Keywords:** IGL1, Grain length, MRNA sequencing (mRNA-seq), Differentially expressed gene (DEG), Phytohormone, Transcription factor (TF)

## Abstract

**Background:**

Although great progress has been made in recent years in identifying novel genes or natural alleles for rice yield improvement, the molecular mechanisms of how these genes/natural alleles regulate yield-associated traits, such as grain length and 1000-grain weight, remain largely unclear. An in-depth understanding of the roles of these genes/natural alleles in controlling yield traits become a necessity to ultimately increase rice yield via novel molecular techniques, such as gene editing.

**Results:**

In this study, the roles of *IGL1*, which was previously identified through a map-based cloning approach, in the regulation of grain length were investigated by overexpressing and knocking out it in the Nipponbare genetic background. Overexpression and knockout of IGL1 (the resulting transgenic lines were hereafter designated IGL1-OE and IGL1-CR lines, respectively) led to elongation and shortening of grains, respectively. To further elucidate the molecular mechanisms behind the IGL1 action, young panicles from IGL1-OE and IGL1-CR lines were subjected to mRNA sequencing. The results showed that both overexpression and knockout of IGL1 all resulted in a large number of upregulated and downregulated differentially expression genes (DEGs) relative to wild-type NPB control lines. A total of 984 DEGs overlapped between upregulated DEGs from IGL1-OE and downregulated DEGs from IGL1-CR; 1146 DEGs were common to downregulated DEGs from IGL1-OE and upregulated DEGs from IGL1-CR. GO term and KEGG pathway analysis revealed that IGL1-upregulated DEGs were associated with extracellular region, protein ubiquitination, cell-wall modification, BR signaling, cell cycle, etc.; by comparison, the IGL1-downregulated DEGs were connected with extracellular region, response to wounding, flavonoid biosynthesis, jasmonic-acid signaling, glucose/sucrose metabolism, etc. Some phytohormone-associated genes (like OsYUCCA4, OsPIN10b, OsBAK1, and OsDLT), TF genes (like OsMADS1 and OsGASR9), grain length-regulating genes (like An-1, GS9, OsIQD14, and TGW2) showed significant upregulation or downregulation in IGL1-OE or IGL1-CR.

**Conclusion:**

Our result clearly demonstrated that IGL1 is an important regulator of grain length, and has profound impacts on genome-wide gene expression, suggesting that it may work together with certain TFs. Overexpression or knockout of IGL1 appears to cause complex expression changes of genes associated with phytohormones, TFs, grain length-regulating factors, which ultimately brings about the grain elongation.

**Supplementary Information:**

The online version contains supplementary material available at 10.1186/s12870-025-06279-2.

## Background

Rice (*Oryza sativa* L.) is the staple food for more than half of the world’s population, and has been used as a model system for the study of monocots. For rice breeding, yield is a very important consideration. Rice yield is largely dictated by three factors: the number of effective tillers per plant, the number of grains per panicle, and the grain weight [1]. Therefore, a better understanding of molecular mechanisms behind the genetic control of these 3 factors will be favorable to the improvement of rice yield. Grain weight is overwhelmingly specified by grain length, grain width and grain thickness, and it is well known that grain length plays a key part in shaping the grain weight. Therefore, identification of genes involved in the regulation of grain length is of great significance for improving grain weight. A large number of studies have shown that some naturally-occurring variant alleles of a gene can exhibit significant positive effects on the regulation of grain length; upon introduction of such alleles into certain cultivars, the grain lengths are considerably increased. Grain Size 3 (GS3), a master regulator of grain length, was discovered from a cultivar named “Minghui 63” by the map-based cloning approach; naturally-occurring loss-of-function *gs3* allele endowed “Minghui 63” with long grains [2]. Further analysis of mechanisms underlying GS3 action revealed that different mutant forms of GS3 have quite different effects on grain size. For example, the mutant allele from “Chuan 7” cultivar, which produced a long truncated protein, gave rise to very short grains, however [3]; by comparison, the mutant allele from “Minghui 63” cultivar, which generated a very short truncated protein, resulted in very long grains [3]. Hence, this suggests that the mutant proteins of different lengths exert quite distinct effects on grain lengths. *OsMADS1* encodes a MADS-box-containing transcription factor (TF) and was discovered to engage in seed-length regulation in rice. The natural truncated allele of *OsMADS1*, named *lgy3*, exhibited significantly elongated grains when it was introgressed into Nipponbare (NPB) genetic background [4]; another study found that upon complete knockout of *OsMADS1*, the knockout lines showed sharply decreased fertility, and grain lengths were appreciably increased [5]. Therefore, it appears that OsMADS1 is a major negative regulator of grain lengths. *Grain Length 3.1* (*GL3.1*), encoded by a major QTL, is a Ser/Thr phosphatase, which belongs to a protein phosphatase kelch (PPKL) family. A natural allele of *GL3.1* from “WY3” cultivar, designated as *GL3.1*^WY3^, coded for a mutant protein with impaired dephosphorylation activity, which thus accelerated cell division and finally resulted in longer grains and higher yields [6]. Surveys of *GL3.1* variants in a variety of rice cultivars revealed that loss-of-function mutations of *GL3.1*, for example in “Nanyangzhan” and “Jizi1560” cultivars, all led to markedly longer grains, indicating that GL3.1 negatively regulates the grain length [6]. GL2, synonymous with OsGRF4, is a transcriptional regulator. The natural mutant allele of *GL2*^RW11^, present in an *indica* cultivar “RW11”, imparted a long-grain phenotype to the “RW11” cultivar; detection of *GL2*^RW11^ transcripts showed that the transcript level was significantly elevated in such a cultivar [7]. Closer analysis revealed that there was two base substitutions (from TC to AA) occurring on the third coding exon of *GL2*^RW11^, which compromised the intensity of miR396 binding to the sequence motif centered around these 2 mutated bases; as a result, the cleavage of *GL2*^RW11^ transcripts by miR396 was correspondingly weakened, thereby leading to increased level of GL2^RW11^ transcripts [7]. GW7 was reportedly a TRM-class protein, and the natural allele *GW7*^TFA^ gave rise to longer grains in comparison with the *GW7*^HJX74^ allele [8]. Sequence analysis revealed that more than ten SNPs and Indels, found on the promoter region, differed between *GW7*^TFA^ and *GW7*^HJX74^ alleles, which led to the expression differences between these two alleles [8]. It is striking that OsSPL16 (encoding a SBP-domain-containing TF, and also known as GW8) was able to repress *GW7* expression by direct binding to the *GW7*’s promoter [8]. Taken together, these studies demonstrate that grain length is regulated by multiple protein factors in a quite complex manner, the factors that involve transcriptional regulators, TFs, phosphatases, TRM-class proteins, etc.

It has been reported that phytohormones play central roles in dominating or modulating rice grain development. Auxin, GA (gibberellin), BR (brassinosteroid) and CTK (cytokinin) are all considered to extensively participate in the regulation of grain length. OsAUX3, an auxin influx carrier, is a member of AUX/LAX family, and functions to transport auxin into the cell in order to keep auxin concentration at a proper level. A natural allele from “9311” cultivar, named *OsAUX3*^9311^, endowed CSSL plants with noticeably longer grains [9]. Sequence analysis of *OsAUX3* between “9311” and NPB cultivars showed that in the *OsAUX3*’s promoter region, there were over 20 SNPs and Indels differing between these two cultivars, which as a result brought about substantially decreased expression levels of the *OsAUX3*^9311^ allele compared with the *OsAUX3*^NPB^ allele [9]. As a consequence, It is more likely that IAA concentration in cells of “9311” was reduced to some extent due to the considerable downregulation of *OsAUX3*^9311^, suggesting that a certain degree of reduction of cellular IAA concentration contributes to grain lengthening, thus resulting in heavier 1000-grain weight [9]. GSK2 is an ortholog of Arabidopsis BIN2, which is a repressor of BR signaling. A recent report showed that overexpression of *GSK2* resulted in BR-deficient plants, including reduced grain sizes, while suppression of *GSK2* expression conferred enhanced BR-signaling phenotypes in rice, as exemplified by enlarged grain sizes [10]. Further investigations revealed that GSK2 was able to phosphorylate DLT, a positive gain-size regulator that functions in BR signaling pathway; phosphorylation of DLT gave rise to diminished DLT activity, and thus knockdown or knockout of *GSK2* activated DLT activity due to impaired phosphorylation on the latter, thereby resulting in enlargement of grains [10]. Arabidopsis BAK1 is capable of forming a heterodimer with BRI1, which can transduce external signals to BZR1 to activate BR signaling [11]. In rice, OsBAK1 plays a similar role as BAK1 does in Arabidopsis, because loss-of-function mutation of *OsBAK1* produces BR-deficient phenotypes, including reduced grain lengths, grain widths, 1000-grain weights, etc. [12], suggesting that OsBAK1 is a positive regulator of grain traits. However, OsBAK2, a paralog of OsBAK1, proved to be a negative regulator of grain length, as knockout of *OsBAK2* led to significantly elongated grains [13], indicating that OsBAK2 regulates the grain length in a manner different from that employed by the OsBAK1. Altogether, these investigations demonstrate that phytohormones have profound influences on grain length, and different signaling-pathway components perform distinct functions in the regulation of grain length.

Except for the above-mentioned transcriptional regulators, TFs, and phytohormones, a variety of cell-wall modifiers also serve wide-ranging functions in fine-tuning grain length. Expansin family constitutes at least 60 members which serve to unlock the network of wall polysaccharides, thus permitting turgor-driven cell enlargement [14]. It has been discovered that a few expansin genes are highly induced in the internodes of deepwater rice plants, and the high levels of expression were closely correlated with rapid elongation of the internodes [14], suggesting that loosening of cell walls is necessary for tissue/organ growth. Xyloglucan endotransglucosylase/hydrolases (XTHs) are able to catalyze the cleavage and molecular grafting of xyloglucan chains, and function in loosening and rearrangement of the cell walls [15]; therefore, XTHs appears to plays important roles in growth and differentiation in plants by regulating cell-wall structures. In an Arabidopsis *acl* mutant*,* XTH9 expression levels were reduced markedly, which brought about short internodal cell lengths [15]. Purified recombinant XTH was found to have enzymatic activity on the extension of isolated cell walls; upon addition of XTH to the isolated epidermis, cell wall extension was significantly increased [16], suggesting the role of XTHs in extending cell walls. In the Arabidopsis *lng1-1D* dominant mutant, the elongated leaves were a result of increased polar cell elongation rather than increased cell proliferation; by comparison, *lng1 lng2* double mutant exhibited decreased leaf lengths as a result of less longitudinal cell elongation [17]. In-depth analysis revealed that the cell elongation in the *lng1-1D* mutant was turgor pressure-driven, and elevated expression of *XTH17* and *XTH24* might perform critical functions in loosening the cell walls in the course of cell elongation [17]. Thus, these results provide a demonstration that XTHs are involved in cell elongation and tissue/organ lengthening through their action in loosening cell walls.

In our previous study, we identified a major QTL, named *IGL1*, by the map-based cloning approach, which was likely to be an important regulator of grain length [1]. In the present study, we analyzed the transcriptome of *IGL1*-overexpressing lines and *IGL1* knockout lines on a genome-wide scale. Our results not only confirmed the positive roles of IGL1 in modulating grain elongation, but also revealed great effects of IGL1 on global gene expression. Overexpression of *IGL1* and knockout of *IGL1* in the NPB genetic background caused 3459 upregulated differentially expressed genes (DEGs) and 3179 downregulated DEGs, respectively, and an assortment of genes implicated in grain-length regulation and/or phytohormone metabolism were significantly affected by changed expression levels of *IGL1*, such as *OsYUCCA6*, *OsYUCCA4*, *OsGA2ox1*, *OsGA3ox1*, *OsGA20ox3*, *OsBAK1*, *OsBZR4*, *OsDLT*, *An-1*, *GS9*, *OsOFP12*, *OsIQD14*, *TGW2*, *OsMADS1*, *OsMAPK6*, etc. Therefore, the results of this study deepen our understanding of IGL1’s effects at the transcriptional level, and lay the groundwork for further investigating how IGL1 participates in the regulation of grain length.

## Methods

### Plant materials and growth conditions

The rice cultivar Nipponbare (*Oryza sativa* L. ssp. *japonica*) was used for transformation in this study. The Nipponbare cultivar was fully sequenced by the International Rice Genome Sequencing Project (https://rice.uga.edu/), and the seeds were obtained from Guo-Liang Wang’s lab in Ohio State University (Columbus, OH). The *IGL1*-OE (for overexpressing *IGL1*) and *IGL1*-CR (for knocking out *IGL1*) lines were generated as described previously [1]. Briefly, to make an *IGL1* overexpression construct, the *IGL1* cDNA sequence (without the stop codon) was PCR-amplified from a reverse-transcribed cDNA pool derived from NPB leaves, and then inserted into a pCAMBIA1300-3 × Flag vector to obtain a final overexpression construct, in which the *IGL1* cDNA was under the transcriptional control of the *35S* promoter; as a consequence, the overexpressed IGL1 protein was fused in-frame with a 3 × Flag tag to produce an IGL1-3 × Flag fusion protein. To make an *IGL1* knockout construct, a sgRNA sequence (19 bp long) was inserted into a CRISPR/Cas9 vector to generate a knockout construct targeting the third coding exon of *IGL1* for editing [1]. Next, the *IGL1* overexpression construct and *IGL1* knockout construct were separately introduced into NPB calli by the Agrobacterium-mediated transformation, and the first-generation (T_0_) transgenic lines were named *IGL1*-OE and *IGL1*-CR lines, respectively. After selfing of *IGL1*-OE lines, qRT-PCR assays were performed to detect the second-generation transgenic plants (T_1_) with overexpression of *IGL1*; meanwhile, PCR-based genotyping were carried out to isolate homozygous *igl1* knockout plants (T_1_ generation) from selfed *IGL1*-CR lines. In order to obtain seeds for phenotypic observations, T_2_ generation seeds harvested from the above-mentioned T_1_ generation plants were germinated, and the one-month-old seedlings, together with their respective non-transgenic wild-type NPB control lines (which were isolated based on the results from the qRT-PCR and genotyping assays), were transplanted to a paddy field on the Baima farm of Nanjing Agricultural University at the end of June, 2024. The planting density was 19.98 cm × 23.31 cm, with one plant per hill. At the booting stage, young panicles of approximately 3 cm length from all genotypes, with 3 biological replicates each, were collected, and then they were immediately frozen in liquid nitrogen and stored at −80 °C for mRNA sequencing (mRNA-seq) analysis.

### Library preparation, mRNA-seq, and data processing

Total RNA, which was isolated from young panicles of the selected *IGL1*-OE, *IGL1*-CR and NPB wild-type control lines, was used to prepare libraries for RNA-seq according to the instructions provided by the library preparation kit (Kangce, China). The resulting libraries were submitted to the Kangce company for mRNA-seq. The obtained raw data were processed by the FastQC (version 0.12.1) software for evaluating data quality. Subsequently, these sequencing data were first filtered by the Trim_galore (version 0.6.10) software; the low-quality reads were discarded, and the reads contaminated with adaptor sequences were trimmed off the raw sequencing data. For analysis of gene expression levels, annotation data of rice reference genome were download from *japonica* reference genome (http://rice.uga.edu/,RGAP) [18], and then the genome indexes were built using Hisat2 software (version 2.2.1) [19]. The resulting clean sequencing data were mapped to the rice reference genome by means of Hisat2 software with default parameters. Reads mapped to the exon regions of each gene were counted by FeatureCounts (version 2.0.6) software [20], and then Counts Per Million (CPM) values were calculated by using the edgeR package (version 4.0.16) [21]. Based on the CPM values, Principal Component Analysis (PCA) was performed by using FactoMineR (version 2.11) and factoextra (version 1.0.7) [22] for reducing background noise, identifying potential classification patterns, and visualizing results.

### Identification of differentially expressed genes (DEGs)

Based on the read counts calculated by FeatureCounts software [20], DEGs between different samples were identified by virtue of the Deseq2 R package (version 1.42.1) [23]. FDR cutoff of 0.05 and fold-change cutoff of 1.5 were used to judge the statistical significance of gene-expression differences. Volcano plots were graphed for visualizing DEGs obtained by the use of the ggplot2 package (version 3.5.1) [24]. If the absolute values of log2(fold change) is greater than log2(1.5), and -log10(*p*-adjust) is greater than -log10(0.05), the value is considered statistically significant. Additionally, the bidirectional stacked bar charts were also illustrated by using ggplot2 to classify the DEGs into distinct gene families/pathway categories that are involved in grain-length regulation.

### Functional enrichment analysis

Gene Ontology (GO) term analysis for DEGs were implemented by Rice Gene Index (RGI) (https://riceome.hzau.edu.cn/,RGI) [25]. The functional annotation of DEGs was classified into three GO classifications: Cellular Component (CC), Biological Process (BP), and Molecular Function (MF). Kyoto encyclopedia of genes and genomes (KEGG) enrichment analysis for DEGs were performed by using the ClusterProfiler R package (version 4.10.1) [26]. The statistical test for GO and KEGG analysis was Fisher’s Exact, and *P* value < 0.05 was considered statistically significant. Based on the log2(fold change) values of the overlapping DEGs, heatmaps were generated by using the R package pheatmap (version 1.0.12) to visualize the degrees of upregulation or downregulation of these overlapping DEGs.

### Measurements of grain lengths, grain widths, and 1000-grain weights

For measurement of grain traits, three independent plants from *IGL1*-OE lines, *IGL1*-CR lines and their respective wild-type NPB control lines were chosen for measuring each grain trait. Approximately 100 fully-filled grains were randomly selected and measured for grain length and width by using a Comi seed scanner (A TS-G-automated analysis system made by Hangzhou Shansheng Testing Technology Co., China). The 1000-grain weight (i.e., the weight of 1000 air-dried grains collected from a single rice plant) was determined with an electronic balance (with an accuracy better than 1 part in 10^3^ g). One-way ANOVA with Duncan’s multiple comparisons was performed to determine statistical significance of differences among different samples.

### Quantitative RT-PCR (qRT-PCR) analysis

RNA samples used for mRNA-seq were also used for qRT-PCR analysis in order to ensure the reliability and repeatability of the results. Total RNA was treated with DNase I (RNase Free) (Vazyme, China) to eliminate possible genomic DNA contamination, and then used to synthesize cDNA in a reverse-transcription reaction by using oligo dT primers (Yeasen, China). The cDNA samples were diluted to 4 ng/μL prior to qRT-PCR assays. Three biological replicates, with three technical replicates each, were performed by using the Hieff® qPCR SYBR Green Master Mix (Yeasen, China) on a 7500 Real-Time PCR machine (Applied Biosystems) according to the manufacturer’s instructions. *OsActin1* served as an internal control. One-way ANOVA with Duncan HSD test for multiple comparisons was carried out to determine statistical significance of differences among different samples. Primer sequences of examined genes were listed in Additional file 2: Table S1.

## Results

### A significant alteration of grain lengths, but not grain widths, caused by overexpression and knockout of IGL1

**Fig. 1:**
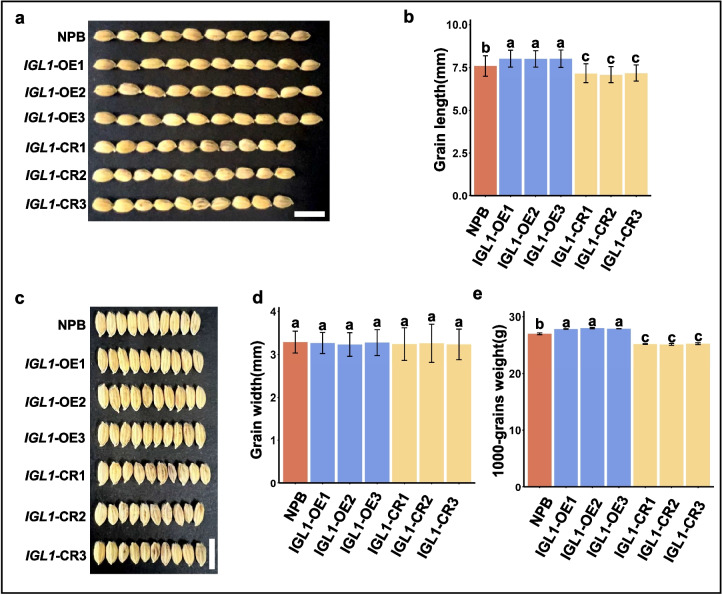
Comparisons of a few grain traits among wild-type NBP control, *IGL1*-OE and *IGL1*-CR lines. Comparisons of grain lengths (**a-b**), grain widths (**c-d**) and 1000-grain weights (**e**) among the three genotypes, wild-type NPB, *IGL1*-OE and *IGL1*-CR lines. Overexpression of *IGL1* in the *IGL1*-OE lines was confirmed by qRT-PCR assays, and homozygous mutations of *IGL1* in the *IGL1*-CR lines were verified by genotyping assays. Values are expressed as means ± SD; for each genotype, approximately 100 seeds from each of 3 plants coming from every genotype were used for measurement, and the obtained data were used for calculating means and standard deviation (SD). Statistical analysis and multiple comparisons were performed by using Duncan's HSD test. Different lowercase letters indicated significant differences between any two samples (*P* < 0.01). For panels (**a**) and (**c**), scale bar = 1 cm

In our previous investigations, we identifies a novel gene, designated as *IGL1* (for *Increased Grain Length 1*), through the map-based approach [1]. The natural allele of *IGL1*, named *igl1*^HD385^ which endowed the upland rice cultivar HD385 with longer grain lengths, was considered to have semi-dominant effects on the grain lengths, because in cases where homozygous *igl1*^HD385^ was introduced into the NPB genetic background, grain lengths of the recipient NPB were significantly increased [1]. To further elucidate the roles of IGL1 in regulating grain length, we generated overexpression lines and knockout lines (created by the CRISPR/Cas9 technique) for *IGL1* in the NPB background. qRT-PCR assays revealed that *IGL1* was significantly upregulated in each of *IGL1* overexpression lines, i.e. *IGL1*-OE1/OE2/OE3 (Additional file 1: Fig. S1a); moreover, a single-base insertion was found in the third coding exon of *IGL1* in each of the CRISPR/Cas9-edited line, i.e. *IGL1*-CR1/CR2/CR3 (Additional file 1: Fig. S1b). It is apparent that overexpression of *IGL1*^NPB^ (the resulting transgenic plants were termed *IGL1*-OE lines) increased grain lengths significantly, and knockout of *IGL1* (the resulting transgenic plants were termed *IGL1*-CR lines), however, decreased grain lengths to a significant degree (Fig. 1a, b). Additionally, altered *IGL1* expression appeared to have little impacts on grain width because either overexpression or knockout of *IGL1* did not result in change of grain width at a significant level (Fig. 1c, d). Correspondingly, the 1000-grain weight of *IGL1*-OE lines was significantly elevated, and that of *IGL1*-CR lines, nevertheless, was significantly reduced (Fig. 1e). These data together demonstrate that IGL1 is indeed a positive regulator for the grain length as well as the 1000-grain weight, and raised expression of a functional *IGL1* or semi-dominant *IGL1* allele (for example, *igl1*^HD385^) gives rise to longer grains and heavier grain weights. Thus, IGL1 appears to be a gene that has good potential for use in improving grain yield.

### Effects of overexpression of IGL1 on transcriptomic profiles

**Fig. 2:**
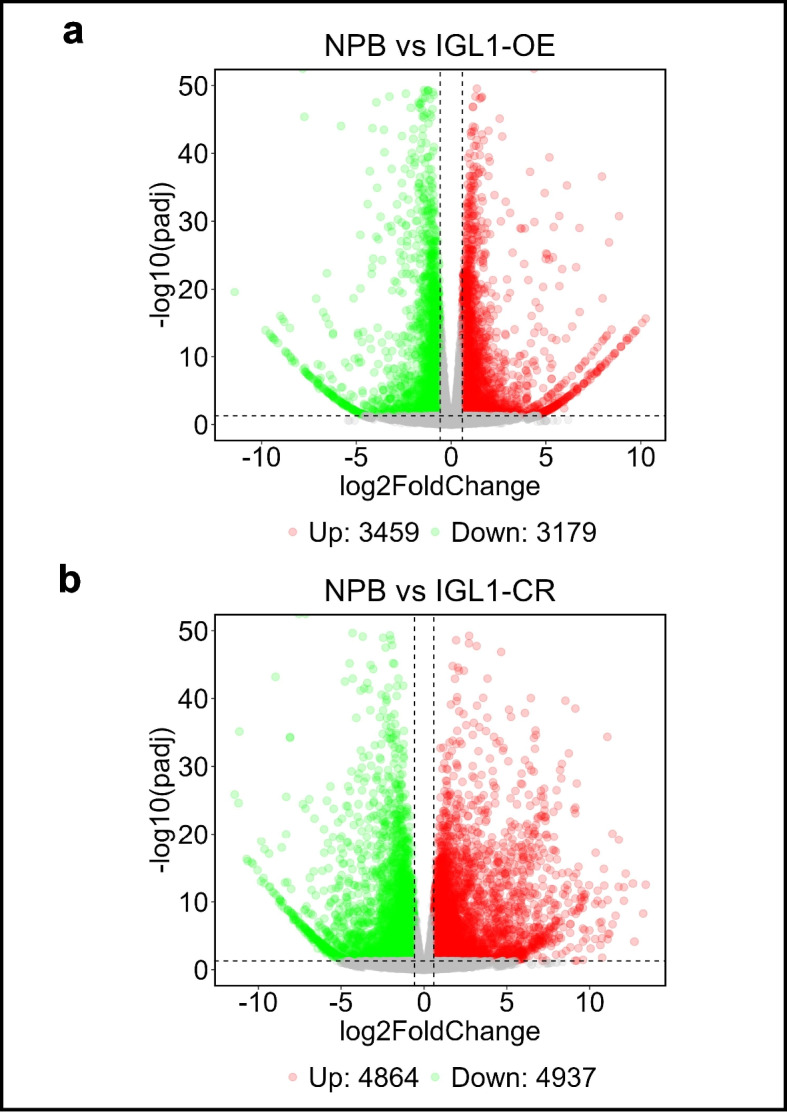
Volcano plots depicting DEGs calculated from *IGL1*-OE and *IGL1*-CR lines relative to their respective wild-type NPB control plants. Significantly upregulated and downregulated DEGs were represented as green and red dots, respectively, in the *IGL1*-OE line versus the wild-type NPB control line (**a**) and in *IGL1*-CR line versus the wild-type NPB control line (**b**). That the absolute value of expression level of a gene in the *IGL1*-OE or *IGL1*-CR line is 1.5-fold or more greater than that of the same gene in the wild-type NPB control (namely, |log2(fold change)|≥ 0.5850) was used as a criterion for judging a significantly upregulated or downregulated DEG. The genes without significant expression changes were shown as grey dots.“Up” and Down” stand for upregulated and downregulated DEGs, respectively

To understand the molecular mechanisms behind the effects of *IGL1* on grain length, we examined the transcriptomic profiles of *IGL1*-OE and *IGL1*-CR lines by the whole-genome mRNA sequencing (mRNA-seq). Principal Component Analysis (PCA) demonstrated a good similarity between biological replicates under the same growth conditions (Additional file 1: Fig. S2a, b). Next, the mRNA-seq data were analyzed and 3459 upregulated DEGs and 3179 downregulated DEGs in the *IGL1*-OE line versus wild-type NPB line (abbreviated to *IGL1*-OE) were detected, respectively, as revealed by a volcano plot (Fig. 2a; Additional file 2: Table S2). The analysis also uncovered a large number of upregulated DEGs (4864) and downregulated DEGs (4937) in the *IGL1*-CR line relative to the wild-type NPB line (abbreviated to *IGL1*-CR) (Fig. 2b). qRT-PCR assays further verified that 7 selected genes, i.e. *OsGA2ox1*, *OsGARS9*, *OsPIN1b*, etc., exhibited expression changes at a statistically significant level (Additional file 1: Fig. S3). These results suggest that an increase in the *IGL1* expression level or complete knockout of *IGL1* makes considerable impacts on the expression of a large number of genes at a genome-wide level.

**Fig. 3:**
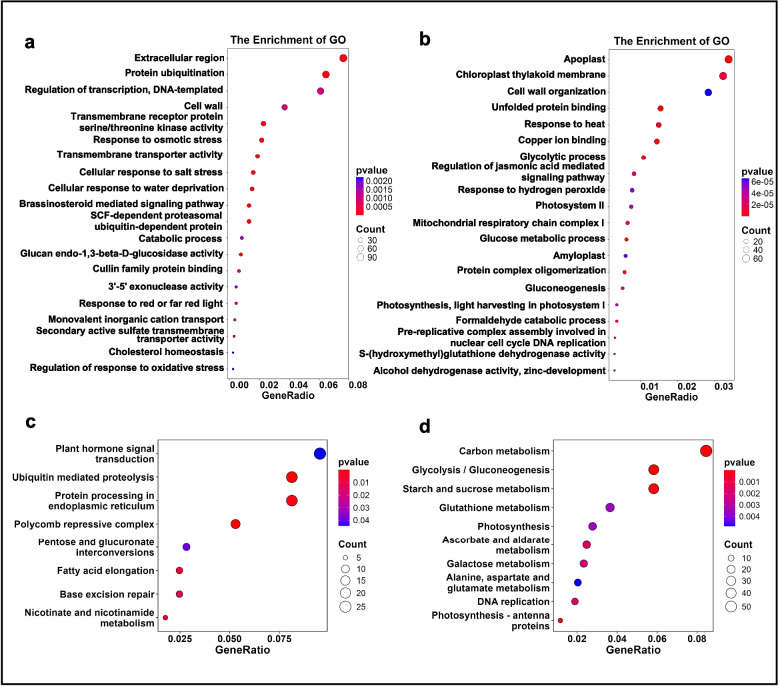
GO term and KEGG enrichment analysis for DEGs from *IGL1*-OE. **a-b** GO term enrichment analysis for upregulated (**a**) and downregulated DEGs (**b**) from *IGL1*-OE. **c-d** KEGG pathway enrichment analysis for upregulated (**c**) and downregulated DEGs (**d**) from *IGL1*-OE. The sizes of dots are proportional to the numbers of DEGs

To get a detailed picture of these DEGs, we performed Gene Ontology (GO) term enrichment analysis to learn about what categories these DEGs fall into. The GO analysis of upregulated DEGs from *IGL1*-OE showed significant enrichment in a total of 20 categories, among which extracellular region, protein ubiquitination, regulation of transcription, cell wall, and transmembrane receptor protein serine/threonine kinase activity were the top 5 classes (Fig. 3a). In addition, DEGs connected with brassinosteroid (BR)-mediated signaling pathway and SCF-dependent proteasomal ubiquitin-dependent protein catabolic process (which is associated with GA and auxin signaling pathways) were also significantly enriched (Fig. 3a). In parallel, GO analysis of downregulated DEGs from the *IGL1*-OE demonstrated that these DEGs were also enriched in 20 categories, of which the top 5 categories were associated with apoplast, chloroplast thylakoid membrane, cell wall organization, unfolded protein binding, and response to heat (Fig. 3b). Besides, GO analysis also revealed significant enrichment in the biological processes linked to the regulation of jasmonic acid (JA)-mediated signaling pathway, glucose metabolic process (which may be functionally relevant to starch accumulation in endosperm), and pre-replicative complex assembly involved in nuclear cell-cycle DNA replication (Fig. 3b).

KEGG analysis was also undertaken to discover what pathways the above-mentioned upregulated and downregulated DEGs fit into. The results showed that, for the upregulated DEGs, a total of 8 pathways were enriched, among which the pathway associated with phytohormone signal transduction was most enriched (Fig. 3c); additionally, a large number of DEGs were also enriched in three pathways, namely, ubiquitin-mediated proteolysis, polycomb repressive complex, fatty acid elongation (Fig. 3c). For the downregulated DEGs, there were 10 pathways enriched, of which carbon metabolism, glycolysis/gluconeogenesis, starch and sucrose metabolism were the top 3 pathways. (Fig. 3d). Altogether, these results demonstrate that overexpression of *IGL1* mainly leads to upregulation of genes associated with the following gene categories: protein ubiquitination, transcriptional regulation, receptor kinase, hormone signaling, SCF-dependent protein degradation, histone modification, and fatty-acid biosynthesis. By comparison, overexpression of *IGL1* chiefly results in downregulation of genes connected with the following gene categories: cell wall organization, JA-mediated signaling, glucose/starch metabolism, and DNA replication.

### Effects of knockout of IGL1 on transcriptomic profiles

**Fig. 4:**
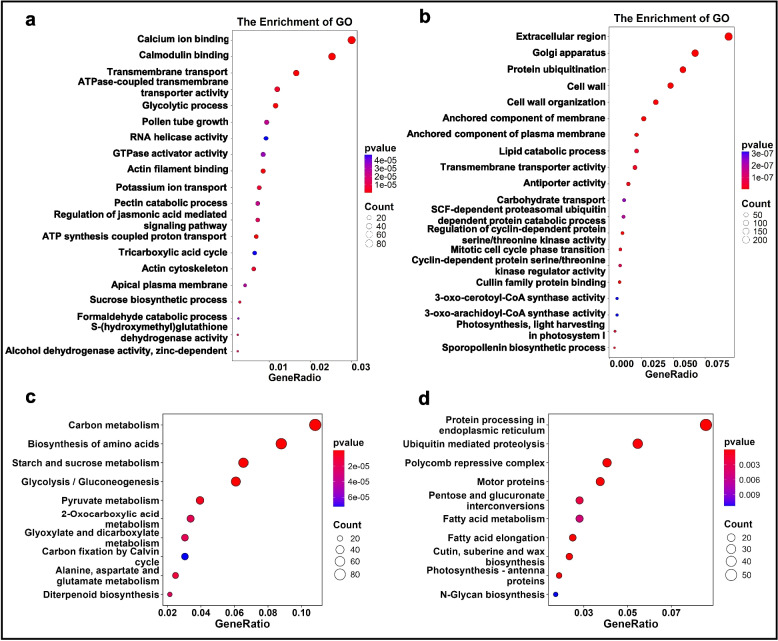
GO term enrichment and KEGG enrichment analysis for DEGs from *IGL1*-CR. **a-b** GO term enrichment analysis for upregulated (**a**) and downregulated DEGs (**b**) from *IGL1*-CR. **c-d** KEGG pathway enrichment analysis for upregulated (**c**) and downregulated DEGs (**d**) from *IGL1*-CR. The sizes of dots are proportional to the numbers of DEGs

The DEGs from *IGL1*-CR were also be subjected to the GO and KEGG enrichment analysis. The upregulated DEGs were significantly enriched in 20 GO terms, among which the top 5 terms were associated with calcium ion binding, calmodulin binding, transmembrane transport, ATPase-coupled transmembrane transporter activity, and glycolytic process (Fig. 4a). There were another 5 GO terms that were seemingly linked to IGL1’s function**—**regulating grain length, which were RNA helicase activity, pectin catabolic process, regulation of JA-mediated signaling pathway, actin cytoskeleton, and sucrose biosynthetic process (Fig. 4a). Similarly, the downregulated DEGs also fell under 20 categories (Fig. 4b). The first 5 GO terms were relevant to extracellular region, Golgi apparatus, protein ubiquitination, cell wall, and cell wall organization. Moreover, another five of the remaining GO terms, i.e. SCF-dependent proteasomal ubiquitin-dependent protein catabolic process, regulation of cyclin-dependent protein serine/threonine kinase activity, mitotic cell cycle phase transition, cyclin-dependent protein serine/threonine kinase, and cullin family protein binding, appeared to be functionally connected to grain-length regulation (Fig. 4b). For the upregulated DEGs, KEGG analysis revealed 10 pathways that these DEGs fall into, with the top 5 categories being carbon metabolism, biosynthesis of amino acids, starch and sucrose metabolism, glycolysis /gluconeogenesis, and pyruvate metabolism, suggesting that loss of IGL1 function principally bring about alteration of carbon metabolism and amino-acid biosynthesis (Fig. 4c). As for the downregulated DEGs, a total of 10 pathways were uncovered by the KEGG analysis, and the pathways, i.e. protein processing in endoplasmic reticulum, ubiquitin mediated proteolysis, polycomb repressive complex, motor proteins, and pentose and glucuronate interconversions constituted the first 5 categories (Fig. 4d). In addition, metabolic process related to fatty acid was overrepresented because the DEGs involved in fatty-acid metabolism and elongation were enriched in their respective categories (Fig. 4d). Taken together, these results indicate that knockout of *IGL1* primarily resulted in upregulation of genes mainly engaging in calcium/calmodulin binding, transmembrane transporter activity, RNA helicase activity, JA-mediated signaling pathway, sucrose and starch metabolism, carbon metabolism, and amino-acid biosynthesis. As a comparison, knockout of *IGL1* mainly gave rise to downregulation of genes largely implicated in protein ubiquitination, cell wall organization and modification, catabolism of component of hormone signaling pathway (undertaken by SCF-dependent proteasome), histone H3K9 methylation, and fatty-acid metabolism.

### Classification of DEGs into distinct gene families/pathway categories that are involved in grain-length regulation

**Fig. 5:**
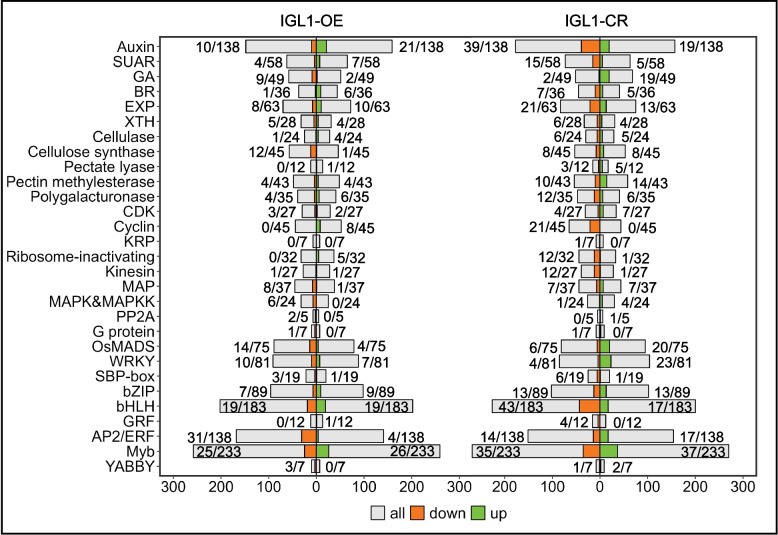
Bidirectional stacked bar charts presenting DEGs that fall into different gene families/pathway categories. The x-axis indicates the numbers of genes, while the y-axis indicates the names of different gene families/pathway categories. The bar chart on the left side shows the numbers of DEGs falling into different gene families/pathway categories in *IGL1*-OE, while the one on the right side presents the numbers of DEGs falling into different gene families/pathway categories in *IGL1*-CR. Green and orange bars depict the relative numbers of upregulated and downregulated DEGs, respectively, and gray bars display the total numbers of genes belonging to each gene family/ pathway category. “Up” and “Down” stand for upregulated DEGs and downregulated DEGs, respectively. The number of DEGs and total number of genes in a gene family/pathway category are shown before and after a slash (/), respectively

Whereas IGL1 serves a function in regulating grain length, we therefore wonder whether there are DEGs falling into the gene families/pathway categories that are implicated in the regulation of grain length. Our analysis revealed that a total of 345 DEGs from *IGL1*-OE fell within 28 gene families/pathway categories (Fig. 5). The top 5 gene families/pathway categories that contained the most DEGs were Myb family [26 out of 233 (26 upregulated DEGs fall into Myb family of 233 member in all); 25 out of 233 (25 downregulated DEGs fall into Myb family of 233 member in all)], bHLH family (19 out of 183 for upregulated DEGs; 19 out of 183 for downregulated DEGs), AP2/ERF family (4 out of 138 for upregulated DEGs; 31 out of 138 for downregualted DEGs), auxin-associated pathway (21 out of 138 for upregulated DEGs; 10 out of 138 for downregulated DEGs), and OsMADS family (4 out of 75 for upregulated DEGs; 14 out of 75 for downregulted DEGs) (Fig. 5). Another two transcription-factor families, WKRY and bZIP, also each involved more than 10 DEGs; that is, WRKY family (consisting of 81 members) contained 7 upregulated and 10 downregulated DEGs, and bZIP family (consisting of 89 members) contained 9 upregulated and 7 downregualted DEGs (Fig. 5). The remaining two classes of TFs, SBP-box and YABBY, involved 1 upregulated and 3 downregulated DEGs for the former, and involved 0 and 3 downregulated DEGs for the latter. In addition, GA- and BR-associated pathways also each contained a number of DEGs: 2 upregulated and 9 downregulated ones in the 49 GA-pathway genes; 6 upregulated and 1 downregulated ones in the 36 BR-pathway genes (Fig. 5). SAUR (for small auxin-up RNA) family included 7 upregulated and 4 downregulated DEGs (Fig. 5). Interestingly, some DEGs fell into cell cycle- and ribosome inactivating-related categories, namely that the former, comprising CDK and cyclin families, contained 10 upregulated and 3 downregulated DEGs, and the latter only 5 upregulated DEGs (Fig. 5). Three families associated with protein phosphorylation/dephosphorylation, MAPK, MAPKK and PP2A, each encompassed a number of DEGs: 0 upregulated and 6 downregulated DEGs for MAPK&MAPKK, 0 upregulated and 2 downregulated DEGs for PP2A. It is striking that 7 cell wall-modifying families, namely, EXP, XTH, cellulose, cellulose synthase, pectate lyase, pectin methylesterase, and polygalacturonase, contained at least one DEG each, with the EXP family possessing the most DEGs, i.e. 10 upregulated and 8 downregulated (Fig. 5). These data together indicate that an increase in IGL1’s activity leads to significant expression changes in some gene families or pathway categories, including the genes encoding TFs (Myb, bHLH, AP2/ERF, OsMADS1, etc.), phytohormone-associated genes (auxin, GA, and BR), phosphorylation/dephosphorylation genes, cell wall-modifying genes, which might give rise to alterations of organ growth and development, as a result of changed phytohormone biosynthesis as well as signaling, transcriptional regulation, and cell wall modifications.

Our analysis of *IGL1*-CR uncovered a total of 568 DEGs falling within 29 gene families/categories associated with grain-length regulation (Fig. 5). The top 5 gene families/pathway categories that included the most DEGs were Myb family (37 out of 233 for upregulated DEGs; 35 out of 233 for downregulated DEGs), auxin-associated pathway (19 out of 138 for upregulated DEGs; 39 out of 138 for downreguled DEGs), bHLH family (17 out of 183 for upregulated DEGs; 43 out of 183 for downregulated DEGs), EXP family (13 out of 63 for upregulated DEGs; 21 out of 63 for downregulated DEGs), and AP2/ERF family (17 out of 138 for upregulated DEGs; 14 out of 138 for downregulted DEGs) (Fig. 5). Like in *IGL1*-OE, GA and BR pathway categories also each contained a number of DEGs; the former encompassed 19 upregulated and 2 downregulated DEGs, and the latter included 5 upregulated and 7 downregulated ones (Fig. 5). Most notably, there existed 5 gene families each involving at least 20 DEGs, i.e. SAUR (totaling 20), pectin methyltransferase (totaling 24), cyclin (totaling 21), OsMADS (totaling 26), and WRKY (totaling 27). These results collectively demonstrate that abolition of *IGL1* function leads to significant expression changes in multiple genes, which belong to tens of gene families/pathway categories, including TF, phytohormone, cell wall modification, cell cycle, etc.

### Analysis of overlapping DEGs that are positively and negatively regulated by IGL1

**Fig. 6:**
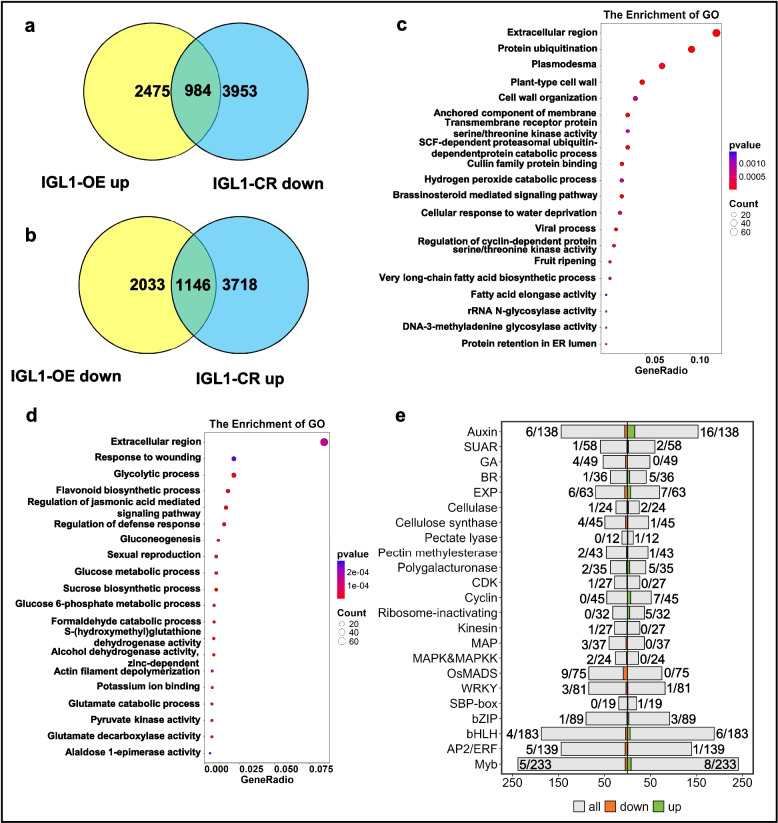
Identification and analysis of overlapping DEGs derived from the overlap between *IGL1*-OE and *IGL1*-CR datasets. **a** Venn diagram illustrating the number of overlapping DEGs (positively regulated by IGL1) derived from the overlap between upregulated DEGs from *IGL1*-OE dataset and downregulated DEGs from *IGL1*-CR dataset. **b** Venn diagram illustrating the number of overlapping DEGs (negatively regulated by IGL1) derived from the overlap between downregulated DEGs from the *IGL1*-OE dataset and upregulated DEGs from the *IGL1*-CR dataset. **c-d** GO term enrichment analysis for the 984 overlapping DEGs shown in (**a**) and for the 1146 overlapping DEGs shown in (**b**). The sizes of dots are proportional to the numbers of DEGs. **e** A bidirectional stacked bar chart depicting the numbers of DEGs that fall into different gene families/pathway categories. To get the numbers of DEGs for drawing this bar chart, all the overlapping DEGs shown in (**a**) (984 plus 1146) were used to overlap with the 23 gene families/pathway categories involved in grain-length regulation. Green and orange bars depict the numbers of upregulated and downregulated DEGs, respectively, and gray bars display the total number of genes belonging to each gene family/pathway category. “Up” and “Down” stand for upregulated and downregulated DEGs, respectively. The number of DEGs and total number of genes in a gene family/pathway category are shown before and after a slash (/), respectively. The x-axis indicates the numbers of genes, while the y-axis indicates the names of different gene families/pathway categories

When we compared the GO terms and KEGG pathway enriched from upregulated DEGs from *IGL1*-OE and the downregulated DEGs from *IGL1*-CR, we found that the enriched GO terms and pathways from both classes of DEGs partly overlapped (Fig. 3a, c and Fig. 4b, d). Likewise, the GO terms and pathways from downregulated DEGs from *IGL1*-OE and the upregulated DEGs from *IGL1*-CR also overlapped to some extent (Fig. 3b,d and Fig. 4a, c). Therefore, we conducted overlap between these four classes of DEGs. As shown in Fig. 6a, there were a total of 984 DEGs overlapping between upregulated DEGs from the *IGL1*-OE dataset and downregulated DEGs from the *IGL1*-CR dataset; meanwhile, 1146 DEGs were common to downregulated DEGs from the *IGL1*-OE dataset and upregulated DEGs from the *IGL1*-CR dataset (Fig. 6b; Additional file 2: Table S3); these DEGs were hereafter referred to as overlapping DEGs. This indicates that the genes that are negatively regulated (directly or indirectly) by IGL1 (1146) are more in number than the ones that are positively regulated (directly or indirectly) by IGL1, implying that IGL1 may play a larger role in repressing gene expression rather than activating gene expression. For the 984 overlapping upregulated DEGs, GO analysis uncovered the significant enrichment for the biological processes associated with extracellular region, protein ubiquitination, plasmodesma, plant-type cell wall, cell wall organization, and so on (Fig. 6c); moreover, significant enrichment of DEGs was also found in the other processes, including SCF-dependent protein catabolic process, BR mediated signaling pathway, regulation of cyclin-dependent protein serine/threonine kinase activity, and fatty-acid biosynthetic process. For the 1146 overlapping dwonregualted DEGs, GO analysis revealed the significant enrichment of DEGs for the biological processes relevant to extracellular region, response to wounding, glycolytic process, flavonoid biosynthetic process, and regulation of JA-mediated signaling pathway etc. (Fig. 6d); furthermore, the DEGs were also enriched for the other processes, such as glucose/sucrose metabolic process and actin-filament depolymerization (Fig. 6d). KEGG analysis demonstrated that, for the overlapping upregulated DEGs, a total of 10 pathways were enriched, among which the pathways associated with ubiquitin-mediated proteolysis, protein processing in endoplasmic reticulum, polycomb repressive complex, fatty-acid biosynthesis and elongation, and tryptophan metabolism (Additional file 1: Fig. S4a). For the overlapping downregulated DEGs, there were 10 pathways in total enriched, which involved the ones connected with carbon metabolism, biosynthesis of amino acids, glycolysis/gluconeogenesis, starch and sucrose metabolism, oxidative phosphorylation, and carbon fixation by calvin cycle, etc. (Additional file 1: Fig. S4b). When we compared the two groups of KEGG pathways, we found that they were totally different: the former was mainly associated with protein degradation, repressive histone modification, fatty-acid metabolism, auxin biosynthesis, while the latter was chiefly connected with carbon metabolism, carbohydrate metabolism, amino acid metabolism, energy and nitrogen metabolism (Additional file 1: Fig. S4a, b); this collectively suggests that IGL1 performs important functions in affecting grain length through simultaneously activating and repressing different classes of genes.

Analysis of all these overlapping DEGs indicated that they fell within 23 gene families/pathway categories [a total of 32 gene families/pathway categories used for this analysis (Additional file 2: Table S4)] connected with grain-length regulation (Fig. 6e). It is noticeable that 22 DEGs in total were enriched in auxin-associated pathway categories, among which 16 DEGs being upregulated and 6 downregulated (Fig. 6e), suggesting that genes participating in the auxin pathway seems to be extensively regulated by IGL1, which in turn implies that such a pathway may be closely involved in grain-length regulation. In addition, there were 4 and 6 DEGs falling into GA and BR pathway categories, respectively (Fig. 6e). A total of 47 DEGs were enriched in the 7 TF families, namely OsMADS1 (9), WRKY (4), SBP-box (1), bZIP (4), bHLH (10), AP2/ERF (6), and Myb (13) (Fig. 6e). Interestingly, the number of DEGs positively regulated by IGL1 (designated as “Up”) (totaling 72) in all the 23 gene families/pathway categories is 11 more than the number of DEGs negatively regulated by IGL1 (designated as “Down”) (totaling 61) (Fig. 6e). These data together suggest that IGL1 has a profound influence on genome-wide gene expression, especially on the expression of genes that engage in grain-length regulation, such as phytohormone-, cell wall modification-, cell cycle-, and TF-associated gene families/pathway categories.

### Impacts of overexpression and knockout of IGL1 on expression levels of overlapping DEGs

**Fig. 7:**
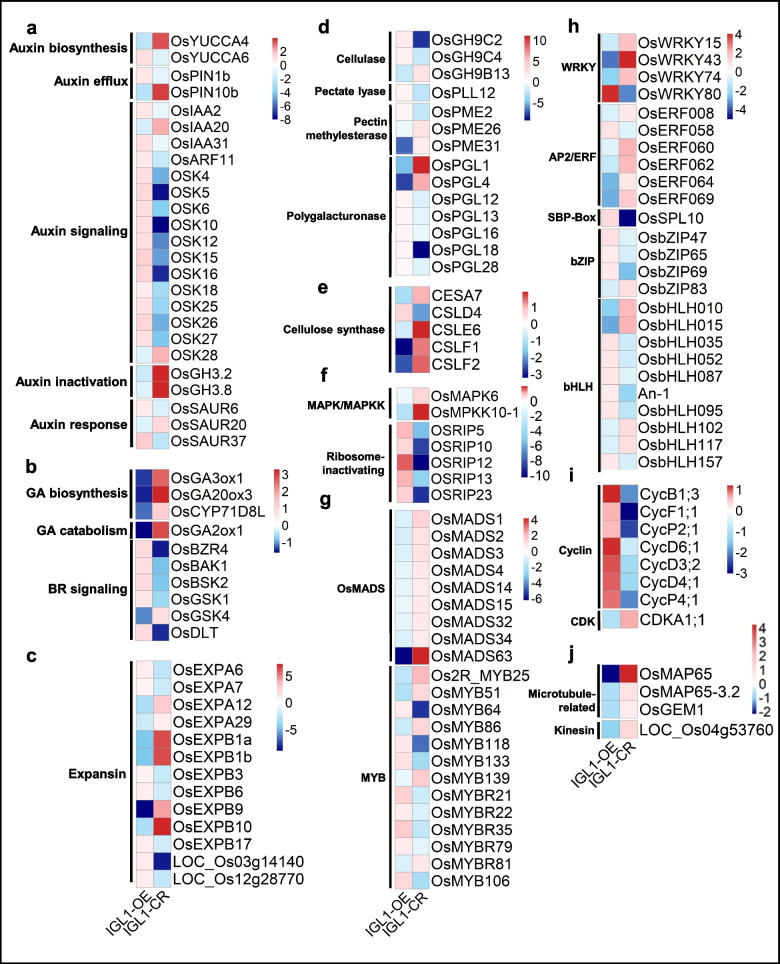
Heatmaps showing the degrees of upregulation/downregulation of the overlapping DEGs from Fig. 6e. The expression levels of a gene in *IGL1*-OE and *IGL1*-CR are shown in the first and second columns, respectively. Red and blue colors represent downregulation and upregulation of DEGs, respectively. The intensities of the colors are reflections of the degrees of gene expression; within each group, the darker the color is, the bigger the fold change is. The color key (blue to red) represents values of [log2(fold change)]

To gain deeper insights into to what extent those overlapping DEGs that fall into the 23 gene families/pathway categories (as shown in Fig. 6e) are modulated by IGL1, we plotted the heatmaps of the DEGs. Among the 22 DEGs involved in the auxin-associated pathway, two genes, *OsYUCCA4* and *OsPIN10b*, which belong to auxin biosynthesis and efflux, respectively, were significantly upregulated in *IGL1*-CR (Fig. 7a). It is clear that the degrees of upregulation/downregulation of these genes in *IGL1*-OE (as shown in the first column) were generally smaller than those of downregulation/upregulated of the same genes in *IGL1*-CR (as shown in the second column) (the intensities of the colors reflect the degrees of gene expression; the darker the color is, the bigger the fold change is) (Fig. 7a), suggesting that loss of IGL1 function makes profound impacts on expression of the majority of genes participating in the auxin pathway (Fig. 7a). For the genes implicated in GA pathway, OsGA3ox1, OsGA20ox3 and GA2ox1 all showed markedly decreased expression in *IGL1*-OE and appreciably increased expression in *IGL1*-CR (Fig. 7b). OsBZR4, a BR-pathway gene, manifested markedly reduced expression in *IGL1*-CR (Fig. 7b). A total of 32 DEGs from 6 cell wall-modifying gene families, i.e. expansin, cellulase, pectate lyase, pectin methylesterase, polygalacturonase and cellulose synthase, exhibited significant expression changes in *IGL1*-OE and *IGL1*-CR (Fig. 7c-e). There were 13 expansin-family genes, which accounted for 20.63% of total expansin genes (63 in total); in *IGL1*-OE, the expression of the 7 expansin genes showed relatively lesser upregulation, while the degrees of downregulation of the remaining 6 genes were markedly higher; by comparison, the degrees of upregulation or downregulation of such genes in *IGL1*-CR were significantly higher when compared with in *IGL1*-OE, especially *OsEXPB1a*, *OsEXPB1b*, *OsEXPB10* and *LOC_Os03g14140* (Fig. 7c). This suggests that loss of *IGL1* has a more significant impact on expression of the 13 genes than overexpression of the *IGL1*. It is striking that the degrees of upregulation or downregulation of the majority of the 5 genes in cellulose-synthase gene family were quite large; moreover, 4 out of 5 were downregulated in *IGL1*-OE and upregulated in *IGL1*-CR (Fig. 7e); this suggests that their expression were substantially affected by IGL1 and may be implicated in grain-length regulation. Remarkably, 5 ribosome-inactivating genes all displayed increased expression in *IGL1*-OE, and decreased expression to a greater degree in *IGL1*-CR (Fig. 7f). TFs are involved in gene’s transcriptional activation or repression. Seven gene families of TFs were significantly upregulated or downregulated in *IGL1*-OE or *IGL1*-CR (Fig. 7g-h). It is surprising that all the 9 MADS-family genes manifested downregulation in *IGL1*-OE and upregulated in *IGL1*-CR, especially *OsMADS63*, which was downregulated in *IGL1*-OE or upregulated in *IGL1*-CR to an exceedingly large extent (Fig. 7g). Given the wide-ranging functions in rice tissue and organ development for OsMADS-family genes, such as floral as well as grain-size control, phytohormone signaling, etc., the downregution of the 9 *OsMADS* genes may exert important influences on multiple development aspects of rice plants to the varying extent. Thirteen *MYB* genes showed significantly expression changes in *IGL1*-OE and *IGL1*-CR (Fig. 7g); a large part of them were upregulated in *IGL1*-OE and downregulated in *IGL1*-CR. For *WRKY* gene family, three genes, *OsWRKY15*, *OsWRKY43* and *OsWRKY74*, were all dowregulated and upregulated in *IGL1*-OE and *IGL1*-CR, respectively (Fig. 7h). As regards *bZIP* gene family, 3 out of 4 genes exhibited upreguation in *IGL1*-OE (Fig. 7h). bHLHs are a class of TFs involved in many respects of plant growth and development. Our analysis uncovered that 6 out of 10 *bHLH* genes were moderately upregulated in *IGL1*-OE (Fig. 7h). It is astonishing that all the 7 *cyclin* genes were upreguated in *IGL1*-OE, especially *CycB1;3*, *CycD6;1*, *CycD3;2*, *CycD4;1* and *CycP4;1*, the degrees of whose upregulation were considerably higher (Fig. 7I). Microtubules and kinesins are known to participate in the process of cell division, and all the 4 genes exhibited downregulation in *IGL1*-OE, particularly *OsMAP65* as being downregulated in *IGL1*-OE and upregulated in *IGL1*-CR to a very large extent (Fig. 7J). Altogether, these data demonstrate that expression alteration of *IGL1* has wide-ranging influences on the expression of multiple families of genes associated with phytohormones (auxin, GA and BR), cell-wall medications, TFs, etc.; overall, knockout of *IGL1* leads to higher degrees of upregulation or downregulation of these genes, when compared with overexpression of *IGL1*.

### Unraveling of influences of expression changes of a set of grain size-associated genes on grain length in IGL1 overexpression and knockout lines

To further understand the molecular mechanisms behind the grain elongation that is observed in the *IGL1*-OE lines, we carefully examined the overlapping DEGs, and then chose a collection of genes that are reportedly involved in seed-length regulation and the genes that are implicated in phytohormone metabolism and homeostasis for further analysis. Finally, two tables were generated: the first one gave a batch of genes that showed upregulation in *IGL1*-OE and downregulation in *IGL1*-CR; the second one gave a collection of genes that exhibited downregulation in *IGL1*-OE and upregulation in *IGL1*-CR (Tables 1 and 2). For the genes in the first table, there were two groups of phytohormone pathway-associated genes, which belonged to auxin and BR categories, respectively (Table 1). As for the auxin category, there were 6 genes, which separately encoded proteins involved in auxin biosynthesis (*OsYUCCA6*), auxin transport (*OsPIN1b*), auxin signaling (*OsARF11*), PIN phosphorylation (*OsWAG1*), auxin-activated TF for establishment of the stem cell niche (*OsPLT7*), and transcription inhibitor of auxin biosynthesis (*OsTIF1*), exhibiting significant expression changes (Table 1). Increased expression of *YUCCA6* (2.02-fold increase) and *OsPIN1b* (1.69-fold increase) may lead to an elevation of auxin concentration and facilitate auxin efflux, thus enhancing auxin signaling [27, 28]. As regards BR category, there were 7 genes showing altered expression: *OsBZR4*, *OsBAK1*, *OsBSK2*, *OsRLCK27*, *OsRLCK184*, *OsGSK1*, and *OsDLT* (Table 1). OsBZR4 is a paralog of OsBZR1 (which is a key BR-activated TF and makes a positive contribution to BR signaling), and may also play positive a role in BR signaling [29]; as a result, its upregulation presumably enhanced BR signaling. OsBAK1 is a co-receptor of BRI1 and also positively contributes to BR signaling; raised expression of *OsBAK1* in *IGL1*-OE might lead to an enhancement of BR signaling [30]. OsBSK2 is reported to positively regulate rice grain size [31]; hence, the heightened transcription of such a gene appeared to be favorable to the enlargement of grain size. OsDLT proves to be a positive regulator of seed size, and it is phosphorylated by OsGSK2, a kinase that is able to interact with OsDLT [10]; thus, the elevated expression of *OsDLT* in *IGL1*-OE was more likely to enhance BR signaling and was helpful in increasing grain size. Although there are many TF-encoding genes found on the list of overlapping DEGs, six of which were thought to be most closely connected with grain-length regulation, i.e. *OsGASR9*, *OsSHR2*, *OsbZIP47*, *OsSPL10*, *An-1*, and *GS9* (Table 1). OsGASR9 was discovered to be a member of plant-specific GA-stimulated transcript family, and functions as a positive regulator of grain size and yield [32]; as a result, it is more than likely that elevation of *OsGASR9* led to increase in grain size. *An-1* encodes a bHLH TF and serves critical functions in regulating awn development, grain size and grain number; overexpression of *An-1* not only lengthens awns, but also gives rise to significant elongation of grain lengths [33]; therefore, the increase of grain lengths observed in *IGL1*-OE lines resulted, at least in part, from the upregulation of *An-1*. GS9 has been reported to be a negative regulator of grain length, that is, upregulation of *GS9*, which was also observed in *IGL1*-OE lines, produced shorter grain in comparison with the wild-type control [34]. A recent report showed that, in fruit, Arabidopsis and rice, the OFP-TRM- and IQD67-mediated pathway separately regulated fruit/grain shapes through integration of phytohormones and microtubule organization [35]; our analysis led to the identification of two genes, *OsOFP12* and *OsIQD14*, which belonged to *OFP* and *IQD76* gene families, respectively (Table 1). Although there was no report on the molecular functions of OsOFP12, OsOFP8 and OsOFP14, both of which are the paralogs of OsOFP12, were discovered to repress the transcriptional activation activity of *GS9* by interacting with GS9 [34]. So we conjecture that OsOFP12 may play some part in repressing other transcriptional activators; upregulation of *OsOFP12*, as observed in *IGL1*-OE, may lead to elongation of rice grain. OsIQD14 was found to be a positive regulator of grain length; its overexpression caused narrow and long grains as a result of changed microtubule alignment [36]. Therefore, increased expression of *OsIQD14* in *IGL1*-OE lines most likely brought about grain lengthening. OsTGW2 encodes a previously unknown RING-type protein with E3 ubiquitin ligase activity, and was found to be a negative regulator of grain width [37]. In *IGL1*-OE lines, *OsTGW2* displayed significantly elevated expression level; however, no significant difference in grain width between grains from *IGL1*-OE lines and grains from NPB control was observed (Table 1). Altogether, these results demonstrate that upregulation of *IGL1* leads to increased expression of a variety of genes that engage in phytohormone-associated pathway (including auxin and BR), transcriptional regulation (like TFs), cell-cycle regulation, modulation of fruit/grain shapes (such as OFP- and IQD-class proteins, TGW2, and SG1), most of which are positive regulators of grain length, thereby resulting in the elongated grains, as observed in *IGL1*-OE lines.

**Table 1:** Selected DEGs that are positively regulated by IGL1

Gene family/pathway category	Gene name	Locus_ID	log2(fold change)	
			**IGL1-OE up**	**IGL1-CR down**
**auxin**	OsYUCCA6	LOC_Os07g25540	1.01	−1.10
	OsPIN1b	LOC_Os02g50960	0.76	−0.91
	OsARF11, OsMP, OsARF5^1^	LOC_Os04g56850	0.70	−1.86
	OsWAG1	LOC_Os03g14840	0.80	−2.16
	OsPLT7	LOC_Os03g56050	0.65	−2.09
	OsTIF1	LOC_Os04g02510	0.59	−0.83
**BR**	OsBZR4	LOC_Os02g13900	0.86	−1.53
	OsBAK1, OsI-BAK1^1^	LOC_Os03g32580	0.83	−0.86
	OsBSK2, OsRLCK308, GLW10^1^	LOC_Os10g42110	0.89	−0.84
	OsRLCK27	LOC_Os01g14510	0.61	−1.25
	OsRLCK184	LOC_Os05g30820	0.87	−2.38
	OsGSK1	LOC_Os01g10840	0.60	−0.63
	OsDLT	LOC_Os06g03710	0.86	−1.47
**TF**	OsGASR9	LOC_Os07g40240	1.01	−1.96
	OsSHR2	LOC_Os03g31880	1.05	−1.78
	OsbZIP47	LOC_Os06g15480	0.93	−0.70
	OsSPL10	LOC_Os06g44860	1.07	−5.07
	An-1	LOC_Os04g28280	0.60	−2.16
	GS9	LOC_Os09g27590	0.76	−1.23
**APC**	OsRING442	LOC_Os02g47870	0.72	−0.59
	OsAPC11	LOC_Os03g19059	0.61	−1.72
**Cell cycle**	OsCDC20	LOC_Os04g51110	0.65	−0.73
**Cell wall-associated**	OsWAK1	LOC_Os01g04409	0.68	−1.41
**OVATE family**	OsOFP12, OsOFP17^1^	LOC_Os04g58820	1.07	−0.95
**IQD67 family**	OsIQD14	LOC_Os08g02250	0.87	−0.99
**Yield-related**	TGW2	LOC_Os02g52550	0.90	−2.66
**Domain of unknown function**	SG1	LOC_Os09g28520	1.19	−1.04

^1^These are different names for the same gene

**Table 2:** Selected DEGs that are negatively regulated by IGL1

Gene family/pathway category	Gene name	Locus_ID	log2(fold change)	
			**IGL1-OE down**	**IGL1-CR up**
**Auxin**	OsYUCCA4	LOC_Os01g12490	−2.15	3.37
	OsPIN10b, OsPIN3b^1^	LOC_Os05g50140	−2.37	3.61
	OsGH3.2	LOC_Os01g55940	−1.73	3.87
	OsGH3.8	LOC_Os07g40290	−1.12	3.94
	OsCAND1	LOC_Os02g07120	−0.66	1.02
**GA**	OsGA2ox1	LOC_Os05g06670	−1.63	2.86
	OsGA3ox1	LOC_Os05g08540	−1.38	2.61
	OsGA20ox3	LOC_Os07g07420	−1.48	3.29
	OsCYP71D8L	LOC_Os02g09220	−1.20	1.24
**BR**	OsGSK4	LOC_Os06g35530	−1.08	0.79
**CTK**	OsCKX4	LOC_Os01g71310	−1.41	1.72
**TF**	OsMADS1	LOC_Os03g11614	−0.88	1.40
	OsMADS2	LOC_Os01g66030	−1.01	0.89
	OsMADS4	LOC_Os05g34940	−0.63	0.66
	Os2R_MYB25, OsMPS^1^	LOC_Os02g40530	−1.66	2.13
	OsbHLH102, OsPIF4, OsPIL11^1^	LOC_Os12g41650	−0.70	1.10
**Kinase**	OsMAPK6	LOC_Os06g06090	−0.65	0.63
	OsMKKK70	LOC_Os01g50410	−1.48	1.31
**Microtubule-associated**	OsMAP65	LOC_Os02g03400	−2.17	4.22
	OsMAP65-3.2	LOC_Os05g47970	−0.73	0.86
**Extra-large G protein**	OsXLG1	LOC_Os06g02130	−0.96	0.89
	OsXLG2	LOC_Os11g10050	−0.73	0.60

^1^These are different names for the same gene

For the genes in the second table, it is prominent that a number of genes fell into 4 phytohormone pathways, i.e. auxin, GA, BR and CTK (Table 2). Noticeably, *OsYUCCA4* (which is responsible for auxin biosynthesis) in *IGL1*-OE lines showed an approximately 4.45-fold reduction in expression compared with in the NPB wild-type line. Moreover, *OsPIN10b* (which is responsible for auxin efflex) was also found downregulated (5.19-fold decrease) in *IGL1*-OE lines, which presumably gave rise to decreased auxin transport and distribution. As for the GA, OsGA2ox1 is in charge of deactivation of active GA; its downregulation (3.10-fold decrease), as observed in *IGL1*-OE line, might lead to an increase of active cellular GA concentration (Table 2). It is interesting that two GA biosynthetic genes, *OsGA3ox1* and *OsGA20ox3*, were also downregulated in *IGL1*-OE and upregulated in *IGL1*-CR. Given the apparent redundancy of *OsGA2ox*, *OsGA3ox* and *OsGA20ox* gene families, it is difficult to determine to what extent the expression changes of the three genes (*OsGA2ox1*, *OsGA3ox1* and *OsGA20ox3*) eventually influences endogenous GA levels and signaling. OsCYP71D8L was discovered to coordinate the homeostasis of GAs and CTKs, and overexpression of such a gene brought about decreased levels of endogenous GAs [38]; thus, downregulation of *OsCYP71D8L* in *IGL1*-OE was more likely to elevate GA levels. Our analysis identified 5 TFs that are implicated in the regulation of grain length (Table 2). Three MADS-family genes, *OsMADS1*, *OsMADS2* and *OsMADS4*, all exhibited downregulation in *IGL1*-OE and upregulation in *IGL1*-CR. OsMADS1, also known as LGY3 or OsLG3b, is a key regulator of grain length, because *lgy3* natural mutation (producing a severely-impaired mutant allele of *OsMADS1*) and knockout of *OsMADS1* all led to longer grains [4, 5]; hence, the increased grain length in *IGL1*-OE line may partially stem from decreased expression of *OsMADS1*. OsMADS2 was reported to perform similar functions as OsMADS1 in modulating grain size [39]; as a consequence, the downregulation of *OsMADS2* in *IGL1*-OE line presumably made a positive contribution to the elongation of grain length. OsMPS belongs to the family of R2R3-type MYB TFs, and was found to be a repressor of expression of a number of expansin genes [40], so the downregulation of *OsMPS* in *IGL1*-OE line may allow the elevated expression of these expansin genes (Table 2). Microtubule-associated proteins are a class of proteins that cross-link the microtubules and are involved in cytokinesis, and *OsMAP65*-defective plants usually displayed obvious cytokinesis failures [41]. Therefore, the impaired cytokinesis, which may be caused by downregulated expression of *OsMAP65*-class genes [for example, OsMAP65 (4.51-fold decrease) and OsMAP65-3.2 (1.66-fold decrease) as observed in *IGL1*-OE line], might give rise to higher ploidy levels, and hence elongated grains (Table 2). Two extra-large G proteins have distinct roles in regulating grain length: loss of OsXLG1 function significantly decreases grain lengths, while disruption of OsXLG2 increases grain lengths to a significant level [42]; thus, the reduced expression of both genes in *IGL1*-OE might make some impacts on the grain length (Table 2).

Taken together, these data revealed that different categories of genes were subject to the regulation of IGL1, which covered the genes associated with phytohormone pathways, TFs, cell-cycle regulation, fruit/grain-shape modulation, grain-length regulation, mitogen-activated protein kinases, microtubule-associated proteins, and extra-large G proteins, etc. It is worth noting that the regulation of phytohormone-pathway genes by IGL1 was quite complex, because some (like *OsYUCCA6*, *OsPIN1b*, *OsBAK1*, and *OsDLT*) were positively regulated by IGL1, but some (like *OsYUCCA4*, *OsPIN10b*, and *OsGA2ox1*) were negatively regulated by IGL1. However, in most cases, either upregulation or downregulation of such genes made positive contributions to the grain elongation, which was probably why overexpression of *IGL1* eventually led to longer grains and knockout of *IGL1* shorter grains, when compared with the wild-type NPB line. With respect to TFs, upregulation (like *OsGASR9* and *An-1*) and downregulation (like *OsMADS1* and *OsMADS2*) of certain genes were undoubtedly favorable to the lengthening of grains. Interestingly, OsOFP12 and OsIQD14, which separately belong to OFP- and IQD-pathway gene families, were all upregulated in *IGL1*-OE, suggesting that the IGL1 may act synergistically with OFP and IQD pathways to render grain longer. Thus, the elongation of grain caused by *IGL1* overexpression is attributable to combined action of many proteins that are involved in different pathways or metabolic processes.

## Discussion

For rice breeding, yield has long been the first consideration. Rice yield is governed by multiple factors, such as tiller number, grain number per panicle, and 1000-grain weight. Considering the limitation of tiller number, increasing grain number per panicle and 1000-grain weight by means of breading strategy become effective measures to improve rice yield. It is well known that 1000-grain weight is inextricably correlated with grain size, which is determined by grain length, grain width and grain thickness. Therefore, many studies have focused on the cloning of new alleles (naturally occurring or mutant alleles) that have positive effects on grain length or width, and characterization of the molecular functions of such alleles in the regulation of grain length/width. In our previous investigations, we identified a novel natural allele, named *IGL1*^HD385^, through the map-based cloning approach, and found that it was a positive modulator of grain length, that is, overexpression of *IGL1*^HD385^ resulted in obviously longer grains, suggesting that this allele was functional [1]. To further dissect the molecular functions of IGL1 in grain-length regulation, we overexpressed and knocked out *IGL1* in the NPB genetic background, respectively (Fig. 1), and subsequently the resulting overexpression lines (designated as *IGL1*-OE lines) and knockout lines (designated as *IGL1*-CR lines) were all subjected to whole-genome mRNA sequencing. Analysis of the mRNA-seq data revealed that there were a large number of genes that were upregulated and downregulated in either *IGL1*-OE lines or *IGL1*-CR lines. For example, 3459 genes were upregulated and 3179 genes were downregulated in the *IGL1*-OE lines relative to the wild-type NPB control line; 4864 genes were upregulated and 4937 genes were downregulated in the *IGL1*-CR lines relative to the wild-type NPB control line (Fig. 2a, b). This suggests that overexpression or knockout of *IGL1* makes substantial impacts on the expression of genes on a genome-wide scale. GO term and KEGG analysis showed that the DEGs that fell into phytohormone pathways, transcription regulation, starch/glucose metabolism, and ubiquitination were found in both *IGL1*-OE and *IGL1*-CR (Figs. 3 and 4), suggesting that these aspects may make considerable influences on grain elongation. Further analysis revealed that there were 984 and 1146 DEGs overlapping between upregulated DEGs in *IGL1*-OE and dowregulated DEGs in *IGL1*-CR and between downregulated DEGs in the *IGL1*-OE and upregulated DEGs in the *IGL1*-CR, respectively (Fig. 6a, b), further confirming that IGL1 has extensive effects on genome-wide gene expression. There were a total of 133 DEGs (which were from all the overlapping DEGs) fitting into 24 gene families/pathway categories that were associated with the regulation of grain length (Fig. 6e), pointing to the fact that overexpression and knockout of *IGL1* leads to expression changes of a large number of DEGs that are involved in wide-ranging metabolic, transcriptional regulation, and signaling pathways. Nevertheless, a domain search with the entire IGL1 protein sequence against relevant protein database was not able to identify any domain related to transcription regulation, indicating that IGL1 does not act as a transcription activator or repressor. Thus, we speculate that IGL1 may work together with certain TFs to modulate gene expression on a genome-wide scale. Moreover, we observed that it was many TFs that were regulated by IGL1, some of which may interact with IGL1. To prove the above speculation, we subsequently performed protein–protein interaction assays between IGL1 and some candidate TFs, and found that IGL1 was indeed able to interact with two proteins, a transcriptional activator and a grain-length regulator. Hence, IGL1 may act in concert with these factors to activate or repress the expression of downstream target genes, which therefore explains why overexpression and knockout of *IGL1* gives rise to extensive expression change of genes at the global level.

Phytohormones have been well known for their wide range of functions for organ growth and development, especially for grain size in rice. Auxin, GA, and BR are all reported to predominantly regulate grain length, but their mode of action is somewhat different. A naturally-occurring active form of auxin, namely IAA, plays important roles in regulating organ size by promoting cell elongation; however, the optimal concentration of IAA for cell elongation is typically in the range of 10^–6^ to 10^–4^ M, and when the concentration is higher than 10^–4^ M, IAA produces inhibitory effects on cell elongation. In fact, it has been reported that the auxin concentrations in spikelet primodia of rice very young inflorescences were quite high relative to in the neighboring tissues, suggesting that the high concentrations of auxin may inhibit the growth of spikelet hulls [43]. Moreover, a recent study also showed that knockout of *OsAUX3*, an auxin influx carrier, brought about elongated grains, further supporting the above-mentioned notion that higher auxin concentrations in spikelets of young inflorescences have inhibitory effects [9]. Therefore, moderate reduction in auxin concentrations in spikelet primodia may contribute to their enlargement. In this study, we found that the expression levels of *OsYUCCA4* and *OsPIN10b* considerably decreased 4.45-fold and 5.19-fold, respectively (Table 2), which more than likely decreased auxin concentrations in the spikelet primodia, and thus led to longer grains. BR is one of the key growth-promoting phytohormones, and four BR-pathway component genes, *OsBZR4*, *OsBAK1*, *OsBSK2*, and *OsDLT*, were all significantly upregulated, which might make positive contributions to grain elongation. Thus, the noticeable grain elongation of *IGL1*-overexpressing lines appears to stem, at least in part, from decreased auxin concentrations together with increased BR signaling in spikelet primodia of young inflorescences.

It is apparent that 13 previously published grain length-regulating genes, i.e. *OsDLT*, *OsGASR9*, *An-1*, *GS9*, *OsIQD14*, *TGW2*, *SG1*, *OsMADS1*, *OsMADS2*, *OsMAPK6*, *OsMKKK70*, *OsXLG1* and *OsXLG2*, showed significantly upregulation or downregulation in *IGL1*-OE or *IGL1*-CR (Tables 1 and 2), demonstrating that IGL1 alone or works in concert with other factors, like TFs, to regulate the expression of these genes. Among them, *OsDLT*, *OsGASR9*, *An-1*, *GS9*, *OsIQD14*, *TGW2* and *SG1* were upregulated in *IGL1*-OE and downregulated in *IGL1*-CR; four out of them, namely, *OsDLT*, *OsGASR9*, *An-1* and *OsIQD14*, had been confirmed to be positive regulators of grain length. Therefore, this means that increased expression of them in *IGL1*-OE are favorable to the elongation of grain. *OsMADS1*, *OsMADS2*, *OsMAPK6*, *OsMKKK70*, *OsXLG1* and *OsXLG2* were downregulated to a significant level in *IGL1*-OE and upregulated in *IGL1*-CR; three out of them, i.e. *OsMADS1*, *OsMADS2* and *OsXLG2*, were proved to be negative regulators of grain length; thus, downregulation of them, as observed in *IGL1*-OE, most likely makes positive contributions to grain elongation. Hence, these results demonstrate that IGL1 regulates grain length also by modulating some particular genes that are responsible specifically for grain-length regulation. So, the grain elongation of *IGL1*-OE lines must partially result from the expression changes of these grain length-regulating genes. Collectively, we eventually come to a conclusion that the elongation of grain length in *IGL1*-OE lines is a result of multiple effects caused by diverse regulatory factors, including transcriptional regulators, phytohormones, and modulators specific to grain length, in a direct or indirect manner.

## Conclusions

Although more than 60 genes associated with grain-length regulation have been cloned so far, the understanding of how the proteins encoded by these genes shape the grain length remains fragmentary at the molecular level. Our study, on the basis of transcriptomic profiles, revealed a complicated regulatory mechanism for the formation of grain length. Our results showed that phytohormones played predominant roles in governing grain length, especially auxin, GA and BR. It seems that a moderate decrease of auxin concentration or transport (as supported by considerable reduction of *OsYUCCA4* and *OsPIN10b* expression in *IGL1*-OE) and an increase of BR signaling (as supported by elevated expression of several BR-signaling genes, e.g. *OsBZR4*, *OsBAK1*, *OsBSK2* and *OsDLT*) made major positive contributions to grain elongation. TFs are also facilitators to promote grain lengthening because their upregulation (as exemplified by *OsGASR9* and *An-1*) or downregulation (as exemplified by *OsMADS1* and *OsMADS2*) was conducive to the lengthening of grains. Besides, two genes, *OsOFP12* and *OsIQD14*, which separately belong to OFP and IQD67 families involved in fruit-shape regulation, were also upregulated in expression under conditions of overexpression of *IGL1*, suggesting that these two regulatory pathways for fruit length also favor the longitudinal grain growth. Therefore, it appears that the elongation of *IGL1*-overexpressing grains is rooted in combinatorial action of different phytohormonal and regulatory pathways.

## Supplementary Information

Additional file 1: Fig S1. Information on mutations produced by CRISPR/Cas9 technique and examination of expression levels of IGL1 in the IGL1 overexpression and knockout lines. (a) Relative expression levels of *IGL1* in the wild-type NPB control, overexpression lines and knockout lines. *IGL1*-OE1–OE3 and IGL1-CR1–CR3 represent *IGL1*-overexpressing and *IGL1* knockout lines, respectively. Data are expressed as means ± SD (*n* = 3). The asterisks indicate significant differences compared with the wild-type NPB (*P< 0.05, **P < 0.01, *t*-test).(b) A diagrammatic representation of the sgRNA-targeted location on the third protein-coding exon. The bases in blue comprise the NGG sequences, and the bases in red are the inserted ones caused by the CRISPR/Cas9-based gene editing technology. NPB, sequence from wild-type Nipponbare; *IGL1*-CR1/CR2/CR3, one sequence from 3 *IGL1* knockout lines. The red arrow points to the position targeted by the sgRNA. Fig S2. Examination of quality of RNA-seq data in *IGL1*-OE and *IGL1*-CR lines. (a-b) Principal Component Analysis (PCA) of the mRNA-seq data derived from *IGL1*-OE, *IGL1*-CR and their respectively wild-type NBP control lines. PCA was performed based on the CPM (Counts Per Million) values to uncover biological variability between different mRNA-seq data and to identify potential clustering or classification patterns among them. For each genotype, young panicles (approximately 3 cm long) from three biological replicates were subjected to mRNA sequencing and the resulting mRNA-seq data were used for the PCA analysis. (c) Examination of expression status of *IGL1* in *IGL1*-OE and *IGL1*-CR lines. The y-axis indicates fold changes of *IGL1* transcript levels in the *IGL1*-OE or *IGL1*-CR line relative to the wild-type NPB control line. Data were calculated from the mRNA-seq data. Fig. S3. Validation of expression levels of a few genes chosen from the transcriptomic profiles by qRT-PCR assays. To verify the validity of transcriptomic profiles, 7 genes were randomly chosen and subjected to qRT-PCR assays for verifying their expression levels in 3-cm-long young panicles. Data are expressed as means of three replicates ± SD, and the asterisks indicate significant differences compared to the NPB under the same growth conditions (*P < 0.05, **P < 0.01, t-test). ns, no significant. Fig. S4. KEGG pathway enrichment analysis for the overlapping DEGs. (a-b) KEGG pathway enrichment analysis for the 984 overlapping DEGs (positively regulated by IGL1) shown in Fig. 6a and for the 1146 overlapping DEGs (negatively regulated by IGL1) shown in Fig. 6b. The sizes of dots are proportional to the numbers of DEGs. Fig. S1. Information on mutations produced by CRISPR/Cas9 technique and examination of expression levels of *IGL1* in the *IGL1* overexpression and knockout lines. (a) Relative expression levels of *IGL1* in the wild-type NPB control, overexpression lines and knockout lines. *IGL1*-OE1–OE3 and *IGL1*-CR1–CR3 represent *IGL1*-overexpressing and *IGL1* knockout lines, respectively. Data are expressed as means ± SD (*n* = 3). The asterisks indicate significant differences compared with the wild-type NPB (*P < 0.05, **P < 0.01, *t*-test). (b) A diagrammatic representation of the sgRNA-targeted location on the third protein-coding exon. The bases in blue comprise the NGG sequences, and the bases in red are the inserted ones caused by the CRISPR/Cas9-based gene editing technology. NPB, sequence from wild-type Nipponbare; *IGL1*-CR1/CR2/CR3, one sequence from 3 *IGL1* knockout lines. The red arrow points to the position targeted by the sgRNA. Fig. S2. Examination of quality of RNA-seq data in *IGL1*-OE and *IGL1*-CR lines. (a-b) Principal Component Analysis (PCA) of the mRNA-seq data derived from *IGL1*-OE, *IGL1*-CR and their respectively wild-type NBP control lines. PCA was performed based on the CPM (Counts Per Million) values to uncover biological variability between different mRNA-seq data and to identify potential clustering or classification patterns among them. For each genotype, young panicles (approximately 3 cm long) from three biological replicates were subjected to mRNA sequencing and the resulting mRNA-seq data were used for the PCA analysis. Fig. S3. Validation of expression levels of a few genes chosen from the transcriptomic profiles by qRT-PCR assays To verify the validity of transcriptomic profiles, 7 genes were randomly chosen and subjected to qRT-PCR assays for verifying their expression levels in 3-cm-long young panicles. Data are expressed as means of three replicates± SD, and the asterisks indicate significant differences compared to the NPB under the same growth conditions (*P< 0.05, **P < 0.01, *t*-test). ns, no significant. Fig. S4. KEGG pathway enrichment analysis for the overlapping DEGs. (a-b) KEGG pathway enrichment analysis for the 984 overlapping DEGs (positively regulated by IGL1) shown in Fig. 6a and for the 1146 overlapping DEGs (negatively regulated by IGL1) shown in Fig. 6b. The sizes of dots are proportional to the numbers of DEGs.

Additional file 2: Table S1. Primers used for the qRT-PCR assays and CRIPSR/Cas9 construct. Table S2. Lists of upregulated and downregulated genes identified from the *IGL1*-OE and *IGL1*-CR lines relative to the wild-type NPB control lines. Table S3. Lists of overlapping genes derived from the overlap between downregulated DEGs from the IGL1-OE dataset and upregulated DEGs from the IGL1-CR dataset, and between upregulated DEGs from the IGL1-OE dataset and downregulated DEGs from the IGL1-CR dataset. Table S4. Lists of 32 gene families used for drawing bidirectional stacked bar charts. 

## Data Availability

The raw mRNA sequencing data were deposited into Sequence Read Archive database with the BioProject under the accession number PRJNA1173336 (http://www.ncbi.nlm.nih.gov/bioproject/1173336).

## Abbreviations

DEG: Differentially expressed gene
GO: Gene Ontology
GS3: Grain Size 3
GL3.1: Grain Length 3.1
IGL1: Increased Grain Length 1
JA: Jasmonic acid
KEGG: Kyoto Encyclopedia of Genes and Genomes
NPB: Nipponbare
PCA: Principal Component Analysis
TF: Transcription factor
XTH: Xyloglucan endotransglucosylase/hydrolase
