# Correction: Transcriptomic analysis Ofers deep insights into the increased grain length 1 (IGL1) regulation of grain length

**DOI:** 10.1186/s12870-025-06645-0

**Published:** 2025-05-06

**Authors:** Liran Sang, Ending Xu, Yan Liu, Tiange Hu, Mengqi Yang, Jiayu Niu, Chong Lu, Yi Zhou, Yifei Sun, Zhaoyu Zhai, Dina Abdulmajid, Peijiang Zhang, Qianqian Wang, Honggui La, Yu Zou

**Affiliations:** 1 College of Life Sciences, Nanjing Agricultural University, Nanjing, Jiangsu 210095 China; 2 Anhui Province Key Laboratory of Rice Genetics and Breeding, Rice Research Institute, Anhui Academy of Agricultural Sciences, Hefei, Anhui 230041 China; 3 College of Artificial Intelligence, Nanjing Agricultural University, Nanjing, 210095 China; 4 Rice Research and Training Centre, Field Crops Research Institute, A.R.C, Sakha, Kafrelsheikh, 33717 Egypt; 5 Department of Breeding, Shandong Peanut Research Institute, Qingdao, 266000 China

**Correction:** ***BMC Plant Biol*** **25** **, 264 (2025)**

10.1186/s12870-025-06279-2

Following publication of the original article [1], the authors identified errors under Affliation 2 and under Author’s Contribution sections. The errors are highlighted in **bold**.

## Current Aff2:

Anhui Province Key Laboratory of Rice Genetics and Breeding, Rice Research Institute, Anhui Academy of Agricultural Sciences**)**, Hefei**aq**, Anhui 230041, China.

## Corrected Aff2: 

Anhui Province Key Laboratory of Rice Genetics and Breeding, Rice Research Institute, Anhui Academy of Agricultural Sciences, Hefei, Anhui 230041, China

## Current Author’s contributions:

L.S., Y.Z. and H.L. conceived the experiments. Y.L. and Z.Z. undertook the data analysis. **L.S.**,** and T.H.** performed the laboratorial experiments. **L.S.**,** Q.W.**,** P.Z. and H.L.** wrote the manuscript. M.Y., C.L., Y.Z., Y.S., and D.A. conducted field experiments. All authors read and approved the final manuscript.

## Corrected Author’s contributions:

L.S., Y.Z. and H.L. conceived the experiments. Y.L. and Z.Z. undertook the data analysis. L.S., T.H., and J.N. performed the laboratorial experiments. L.S., E.X., Q.W., P.Z. and H.L. wrote the manuscript. M.Y., C.L., Y.Z., Y.S., and D.A. conducted field experiments. All authors read and approved the final manuscript.

## Current funding:

This work was supported by grants from Open Research Fund Program of Anhui Province Key Laboratory of Rice Genetics and Breeding (SDKF-2023–04) to Q. Wang, and by grants from Jiangsu Agriculture Science and Technology Innovation Fund (JASTIF) [CX(22)3156] to H. La.

## Corrected funding:

This work was supported by grants from Open Research Fund Program of Anhui Province Key Laboratory of Rice Genetics and Breeding (SDKF-2023–04) to Q. Wang, from Young Talent Project of Anhui Academy of Agricultural Sciences (QNYC-202504) to Y. Zou, and from Jiangsu Agriculture Science and Technology Innovation Fund (JASTIF) [CX(22)3156] to H. La.

The corrections do not compromise the validity of the conclusions and the overall content of the article. The author group has been updated above and the original article [1] has been corrected.

## References

[1] Sang L, Xu E, Liu Y, et al. Transcriptomic analysis offers deep insights into the *Increased grain length 1* (*IGL1*) regulation of grain length. BMC Plant Biol. 2025;25:264. 10.1186/s12870-025-06279-2. 40011803 10.1186/s12870-025-06279-2 PMC11866874

