# Supplementary material for: Transcriptomic analysis offers deep insights into the Increased Grain Length 1 (IGL1) regulation of grain length
Source: BMC Plant Biol. 2025 Feb 27;25:264. doi: 10.1186/s12870-025-06279-2 (PMC11866874; doi:10.1186/s12870-025-06279-2)
Supplement: Supplementary file 1 — Additional file 1: Fig S1. Information on mutations produced by CRISPR/Cas9 technique and examination of expression levels of IGL1 in the IGL1 overexpression and knockout lines. (a) Relative expression levels of IGL1 in the wild-type NPB control, overexpression lines and knockout lines. IGL1-OE1–OE3 and IGL1-CR1–CR3 represent IGL1-overexpressing and IGL1 knockout lines, respectively. Data are expressed as means ± SD (n = 3). The asterisks indicate significant differences compared with the wild-type NPB (*P< 0.05, **P < 0.01, t-test).(b) A diagrammatic representation of the sgRNA-targeted location on the third protein-coding exon. The bases in blue comprise the NGG sequences, and the bases in red are the inserted ones caused by the CRISPR/Cas9-based gene editing technology. NPB, sequence from wild-type Nipponbare; IGL1-CR1/CR2/CR3, one sequence from 3 IGL1 knockout lines. The red arrow points to the position targeted by the sgRNA. Fig S2. Examination of quality of RNA-seq data in IGL1-OE and IGL1-CR lines. (a-b) Principal Component Analysis (PCA) of the mRNA-seq data derived from IGL1-OE, IGL1-CR and their respectively wild-type NBP control lines. PCA was performed based on the CPM (Counts Per Million) values to uncover biological variability between different mRNA-seq data and to identify potential clustering or classification patterns among them. For each genotype, young panicles (approximately 3 cm long) from three biological replicates were subjected to mRNA sequencing and the resulting mRNA-seq data were used for the PCA analysis. (c) Examination of expression status of IGL1 in IGL1-OE and IGL1-CR lines. The y-axis indicates fold changes of IGL1 transcript levels in the IGL1-OE or IGL1-CR line relative to the wild-type NPB control line. Data were calculated from the mRNA-seq data. Fig. S3. Validation of expression levels of a few genes chosen from the transcriptomic profiles by qRT-PCR assays. To verify the validity of transcriptomic profiles, 7 genes were rando [file 12870_2025_6279_MOESM1_ESM.docx]

**Supplementary Information**

The following materials are available in the online version of this article.

**Additional file 1:** **Fig S1.** Information on mutations produced by CRISPR/Cas9 technique and examination of expression levels of IGL1 in the IGL1 overexpression and knockout lines. **(a)** Relative expression levels of *IGL1* in the wild-type NPB control, overexpression lines and knockout lines. *IGL1*-OE1–OE3 and IGL1-CR1–CR3 represent *IGL1*-overexpressing and *IGL1* knockout lines, respectively. Data are expressed as means ± SD (n = 3). The asterisks indicate significant differences compared with the wild-type NPB (**P* < 0.05, ***P* < 0.01, *t*-test).**(b)** A diagrammatic representation of the sgRNA-targeted location on the third protein-coding exon. The bases in blue comprise the NGG sequences, and the bases in red are the inserted ones caused by the CRISPR/Cas9-based gene editing technology. NPB, sequence from wild-type Nipponbare; *IGL1*-CR1/CR2/CR3, one sequence from 3 *IGL1* knockout lines. The red arrow points to the position targeted by the sgRNA. **Fig S2.** Examination of quality of RNA-seq data in *IGL1*-OE and *IGL1*-CR lines. **(a-b)** Principal Component Analysis (PCA) of the mRNA-seq data derived from *IGL1*-OE, *IGL1*-CR and their respectively wild-type NBP control lines. PCA was performed based on the CPM (Counts Per Million) values to uncover biological variability between different mRNA-seq data and to identify potential clustering or classification patterns among them. For each genotype, young panicles (approximately 3 cm long) from three biological replicates were subjected to mRNA sequencing and the resulting mRNA-seq data were used for the PCA analysis. **(c)** Examination of expression status of *IGL1* in *IGL1*-OE and *IGL1*-CR lines. The y-axis indicates fold changes of *IGL1* transcript levels in the *IGL1*-OE or *IGL1*-CR line relative to the wild-type NPB control line. Data were calculated from the mRNA-seq data. **Fig. S3.** Validation of expression levels of a few genes chosen from the transcriptomic profiles by qRT-PCR assays. To verify the validity of transcriptomic profiles, 7 genes were randomly chosen and subjected to qRT-PCR assays for verifying their expression levels in 3-cm-long young panicles. Data are expressed as means of three replicates ± SD, and the asterisks indicate significant differences compared to the NPB under the same growth conditions (*P < 0.05, **P < 0.01, t-test). ns, no significant. **Fig. S4.** KEGG pathway enrichment analysis for the overlapping DEGs. (**a-b)** KEGG pathway enrichment analysis for the 984 overlapping DEGs (positively regulated by IGL1) shown in Fig. 6a and for the 1146 overlapping DEGs (negatively regulated by IGL1) shown in Fig. 6b. The sizes of dots are proportional to the numbers of DEGs.

**Additional file 1: Fig. S1. Information on mutations produced by CRISPR/Cas9 technique and examination of expression levels of *IGL1* in the *IGL1* overexpression and knockout lines**

**(a)** Relative expression levels of *IGL1* in the wild-type NPB control, overexpression lines and knockout lines. *IGL1*-OE1–OE3 and *IGL1*-CR1–CR3 represent *IGL1*-overexpressing and *IGL1* knockout lines, respectively. Data are expressed as means ± SD (n = 3). The asterisks indicate significant differences compared with the wild-type NPB (**P* < 0.05, ***P* < 0.01, *t*-test).

**(b)** A diagrammatic representation of the sgRNA-targeted location on the third protein-coding exon. The bases in blue comprise the NGG sequences, and the bases in red are the inserted ones caused by the CRISPR/Cas9-based gene editing technology. NPB, sequence from wild-type Nipponbare; *IGL1*-CR1/CR2/CR3, one sequence from 3 *IGL1* knockout lines. The red arrow points to the position targeted by the sgRNA.

**Additional file 1: Fig. S2. Examination of quality of RNA-seq data in *IGL1*-OE and *IGL1*-CR lines**

**(a-b)** Principal Component Analysis (PCA) of the mRNA-seq data derived from *IGL1*-OE, *IGL1*-CR and their respectively wild-type NBP control lines. PCA was performed based on the CPM (Counts Per Million) values to uncover biological variability between different mRNA-seq data and to identify potential clustering or classification patterns among them. For each genotype, young panicles (approximately 3 cm long) from three biological replicates were subjected to mRNA sequencing and the resulting mRNA-seq data were used for the PCA analysis.

**Additional file 1: Fig. S3. Validation of expression levels of a few genes chosen from the transcriptomic profiles by qRT-PCR assays**

To verify the validity of transcriptomic profiles, 7 genes were randomly chosen and subjected to qRT-PCR assays for verifying their expression levels in 3-cm-long young panicles. Data are expressed as means of three replicates ± SD, and the asterisks indicate significant differences compared to the NPB under the same growth conditions (**P* < 0.05, ***P* < 0.01, *t*-test). ns, no significant.

**Additional file 1: Fig. S4. KEGG pathway enrichment analysis for the overlapping DEGs**

(**a-b**) KEGG pathway enrichment analysis for the 984 overlapping DEGs (positively regulated by IGL1) shown in Fig. 6a and for the 1146 overlapping DEGs (negatively regulated by IGL1) shown in Fig. 6b. The sizes of dots are proportional to the numbers of DEGs.
