# Supplementary material for: Transcriptomic analysis offers deep insights into the Increased Grain Length 1 (IGL1) regulation of grain length
Source: BMC Plant Biol. 2025 Feb 27;25:264. doi: 10.1186/s12870-025-06279-2 (PMC11866874; doi:10.1186/s12870-025-06279-2)
Supplement: Supplementary file 2 — Additional file 2: Table S1. Primers used for the qRT-PCR assays and CRIPSR/Cas9 construct. Table S2. Lists of upregulated and downregulated genes identified from the IGL1-OE and IGL1-CR lines relative to the wild-type NPB control lines. Table S3. Lists of overlapping genes derived from the overlap between downregulated DEGs from the IGL1-OE dataset and upregulated DEGs from the IGL1-CR dataset, and between upregulated DEGs from the IGL1-OE dataset and downregulated DEGs from the IGL1-CR dataset. Table S4. Lists of 32 gene families used for drawing bidirectional stacked bar charts. [file 12870_2025_6279_MOESM2_ESM.docx]

**Additional file 2:** **Table S1.** Primers used for the qRT-PCR assays and CRIPSR/Cas9 construct**. Table S2.** Lists of upregulated and downregulated genes identified from the *IGL1*-OE and *IGL1*-CR lines relative to the wild-type NPB control lines. **Table S3.** Lists of overlapping genes derived from the overlap between downregulated DEGs from the IGL1-OE dataset and upregulated DEGs from the IGL1-CR dataset, and between upregulated DEGs from the IGL1-OE dataset and downregulated DEGs from the IGL1-CR dataset. **Table S4.** Lists of 32 gene families used for drawing bidirectional stacked bar charts.
